# LDH-Based Voltammetric Sensors

**DOI:** 10.3390/mi15050640

**Published:** 2024-05-10

**Authors:** Domenica Tonelli, Matteo Tonelli, Stefano Gianvittorio, Andreas Lesch

**Affiliations:** 1 Dipartimento di Chimica Industriale “Toso Montanari”, Università di Bologna, Via Piero Gobetti 85, 40129 Bologna, Italy; stefano.gianvittorio@unibo.it (S.G.); andreas.lesch@unibo.it (A.L.); 2 ANRT—Association Nationale de le Reserche et de la Technologie, 33, Rue Rennequin, 75017 Paris, France; tonelli@anrt.asso.fr

**Keywords:** layered double hydroxides, modified electrodes, voltammetric sensors, electrocatalysis, anion exchangeability

## Abstract

Layered double hydroxides (LDHs), also named hydrotalcite-like compounds, are anionic clays with a lamellar structure which have been extensively used in the last two decades as electrode modifiers for the design of electrochemical sensors. These materials can be classified into LDHs containing or not containing redox-active centers. In the former case, a transition metal cation undergoing a reversible redox reaction within a proper potential window is present in the layers, and, therefore, it can act as electron transfer mediator, and electrocatalyze the oxidation of an analyte for which the required overpotential is too high. In the latter case, a negatively charged species acting as a redox mediator can be introduced into the interlayer spaces after exchanging the anion coming from the synthesis, and, again, the material can display electrocatalytic properties. Alternatively, due to the large specific surface area of LDHs, molecules with electroactivity can be adsorbed on their surface. In this review, the most significant electroanalytical applications of LDHs as electrode modifiers for the development of voltammetric sensors are presented, grouping them based on the two types of materials.

## 1. Introduction

Surface modification is a very useful technique in electroanalytical chemistry. The major reasons to produce modified electrodes (MEs) are related to the enhancement of the performance in the presence of thin films of various organic or inorganic modifiers. The main effects connected to the modification of the working electrode surface of an electrochemical sensing platform are as follows: (i) the increase in sensitivity due to a larger electroactive area, (ii) the improved selectivity due to the chemical or electrochemical properties of the modifier, (iii) the possibility to preconcentrate or adsorb the analyte, (iv) the enhanced anti-interference effect in complex matrices, and (v) the reduction or elimination of the electrode fouling, just to name the most important ones [1,2]. The electrocatalytic properties are one of the distinguishing features of MEs used in electroanalytical chemistry [3]. Layered double hydroxides (LDHs), also named hydrotalcite-like compounds, are clays with two-dimensional nanostructures, which consist of brucite-like sheets which are positively charged due to the partial substitution of Mg^2+^, or, in general, a bivalent cation, with a trivalent one. Anions and water molecules are located inside the interlayers, the former to ensure the electroneutrality of the material. The chemical formula of LDHs can be expressed as [M(II)_1−x_M(III)_x_(OH)_2_]^x+^(A^n−^)_x/n_·yH_2_O, where A^n−^ is the interlayer anion, and x is a stoichiometric coefficient which usually ranges between 0.22 and 0.3. The properties of LDHs are easily tunable as many cations and several anions can be introduced in the brucitic layers and interlayers, respectively, giving rise to a huge number of synthetic LDHs [4]. They are also known as anionic clays due to their capacity to act as anionic exchangers. LDHs have been attracting more and more the interest of electroanalytical chemists in the last two decades as electrode surface modifiers, owing to a lot of advantages, such as their large surface area, ease of preparation, low cost, tunable composition, excellent biocompatibility, and electrocatalytic properties. Thus, their application in the construction of electrochemical sensors and biosensors has been widely reported [1,5], and it can be expected that their application fields will expand even more. The LDHs containing transition metals like Ni, Co, or Mn, which are able to undergo reversible redox reactions [6,7], and, therefore, to act as electron transfer mediators, are of great significance for catalysis, energy storage, and sensing [8,9]. In such a case, the electrocatalytic mechanism responsible for analyte detection can be represented by the following reactions:

[M_red_] − LDH + OH^−^ ⇄ [M_ox_ − OH] − LDH + e^−^ (1)

n[M_ox_ − OH] − LDH + reduced analyte → n[M_red_] − LDH + oxidized analyte (2)

where [M_red_] − LDH and [M_ox_ − OH] − LDH represent the redox-active centers of an LDH in their reduced and oxidized states, respectively.

However, LDHs are weakly conductive solids, which is a feature that can limit their electrochemical performance. To improve their conductivity, the incorporation of metal nanoparticles, such as Au, Pt, and Ag, into LDHs is a useful strategy which further enhances their electrocatalytic properties [5], as well as the synthesis of composites based on LDHs and carbon nanomaterials, which recently have been proposed as electrode modifiers for the quantification of molecules of biochemical interest [10,11]. Alternatively, non-conductive LDHs, i.e., LDHs not containing a redox-active cation in the brucitic layer, can be involved in an electrocatalytic effect by exchanging the interlayer anion coming from the synthesis with (i) a negatively charged metal complex such as [Fe(CN)_6_]^3−^, [Mo(CN)_8_]^4−^, and [IrCl_6_]^2−^ undergoing a reversible electron transfer [12], or with ii) an electroactive organic molecule bearing an anionic group such as nitrobenzene sulfonate, anthraquinone monosulfonate or disulfonate, and 2,2′-azinobis(3-ethylbenzothiazoline-6-sulfonate) (ABTS) [13]. Furthermore, due to the large specific surface area of LDHs, molecules with electroactivity can be adsorbed on these materials. As an example, the Fe(III)/Fe(II) couple in nickel hexacyanoferrate immobilized onto the LDH surface has recently been proposed for the development of a voltammetric sensor for doxorubicin [14]. Other layered nanomaterials have been extensively used for the modification of conductive supports with the aim to develop electrochemical sensors, displaying high sensitivities. Among them, graphene (G) has many advantages compared to others because of its excellent mechanical properties, large surface area, high electrical conductivity, high biocompatibility, and excellent electrochemical activity. To date, graphene is still an expensive material, and it is very often substituted by reduced graphene oxide (rGO), since GO is easily produced at a low cost from the oxidation of graphite, and, thereafter, easily reduced to rGO. Many sensors and biosensors based on G or related materials have been reported in the literature especially for the determination of molecules of clinical and biochemical interest [15].

Since the discovery of mechanically exfoliated graphene in 2004, the attention of material scientists was directed towards the discovery of new kinds of 2D nanomaterials, and, in 2011, the first MXene was discovered, by exfoliating pristine Ti_3_AlC_2_ phases with HF. With the denomination of MXene, we mean layered structures made out of transition metal carbides, nitrides, and carbonitrides, with properties similar to graphene, and, in addition, the ease of synthesis in water, which have found brilliant applications in analytical chemistry [16]. MXenes possess abundant surface functional groups (especially –OH) that make them ideal carriers for biomolecules like enzymes, antibodies, or aptamers for the construction of electrochemical biosensors [17].

Compared to the layered materials just mentioned, LDHs display the enormous advantage of being made of elements which are abundant in the earth, not toxic, and environmentally friendly, and, above all, their synthesis is very simple, and can last only a few minutes if electrosynthesis is employed [1]

In this paper review, we review some of the significant electroanalytical applications of LDHs as electrode modifiers for the development of voltammetric sensors, highlighting those based on the most peculiar properties of these fascinating materials.

## 2. LDHs Containing Redox-Active Metal Centers

### 2.1. Ni- and/or Co-Based LDHs

Among the wide range of redox-active metal centers that can be implemented in LDHs, some metal combinations have found particular interest over decades. For instance, in 1999, our group proposed especially Ni/Al-LDH-modified electrodes for the electrocatalytic oxidation of methanol and ethanol in strongly alkaline solutions, by exploiting the presence of Ni cations [18]. The Ni cations undergo a quasi-reversible redox reaction converting Ni^2+^ to Ni^3+^, and, in a second step, the Ni^3+^ centers oxidize the analytes that diffuse inside the LDH structure and possess standard redox potentials lower than that of the Ni^3+^/Ni^2+^ couple. As a result, Ni^2+^ is regenerated, because an electron is transferred from methanol or ethanol during their oxidation to Ni^3+^. The Ni^3+^/Ni^2+^ couple acts, therefore, as a redox mediator for electrons from the electrode to the analyte. In such a way, a decrease in the overpotential, which is necessary in order to oxidize the analyte in the absence of the redox mediator, occurs, and the analytical signal, which is correlated to the analyte concentration, is the increase in Ni^2+^ oxidation current [1]. Taking as an example the LDHs containing Ni as the redox-active center, the charge transport inside the clay can be thought to be due to a mixed mechanism involving an “electron hopping” along the brucitic layers, which is related to the inner redox reaction between oxidized and reduced forms of the Ni^3+^/Ni^2+^ couple, and the OH− intercalation/deintercalation process inside/outside the interlayers to ensure the LDHs electroneutrality. The conduction mechanism was studied by using electrochemical impedance spectroscopy (EIS) from which it was demonstrated that the electronic conductivity of the LDHs material is dependent on the applied electrode potential, while the resistance related to the ionic charge transfer, when an anodic potential is applied, is almost potential-independent [19]. It was concluded that, at the potentials for which Ni centers are partially oxidized, the OH^−^ concentration has a strong influence on the overall conductivity of the material, which increases as the pH increases. As mentioned, vide supra, the selection of metal cations influences the electrochemical performance of LDHs as electrodes. For instance, the substitution of Ni with Co does not significantly alter the characteristics of the modified electrodes, but Co^2+^ is oxidized at a slightly lower potential than Ni^2+^, and this feature introduces a selectivity in the electro-oxidation of molecules containing hydroxyl groups. In particular, Co/Al-LDH-modified electrodes do not display electrocatalytic activity towards monohydric compounds, and, consequently, only molecules containing more than one hydroxyl functional group can be oxidized when the redox mediator is the Co^3+^/Co^2+^ couple.

In the following, some recent applications considering transition-metal-based LDHs are described. A voltammetric sensor for the determination of L-cysteine (Cys) has recently been reported exploiting a pencil graphite electrode (PGE) which was modified with a Co/Al LDH, which was electrochemically synthesized to obtain a disposable and low-cost device [20]. Working in the optimal conditions, i.e., 0.1 M NaOH at a scan rate of 10 mV s^−1^, the oxidation peak potential for the electrocatalytic Cys oxidation was near 0.15 V, vs. SCE. The sensor displayed a linear response in the 100 pM to 0.1 μM Cys concentration range and a 100 pM detection limit, using differential pulse voltammetry (DPV) as the analytical technique. Among possible interferents, molecules such as glucose, citric acid, uric acid, and ascorbic acid were tested, and only ascorbic acid presented a serious interference effect. The PGE was also used to quantify Cys in acetylcysteine effervescent tablets, in order to demonstrate a real sample analysis.

It has been theoretically and experimentally demonstrated, taking into account especially the water and methanol oxidation reactions, that substituting Al with Fe in the brucitic layers of Ni-based LDHs promotes electron transfer, thus increasing the electrocatalytic activity of Ni(III) centers [4,21]. One of the most quoted hypotheses is that Fe could enhance the activity of Ni ions, favoring the delocalization of electrons through a Ni–Fe partial charge-transfer activation process occurring throughout the LDH film.

In another example, Farithkhan and John reported the growth of 3D coral-like Ni/Fe LDHs on the inner walls of the interconnected microchannels of nitrogen carbonized wood (NCW) in order to develop a disposable sensor for the sensitive determination of urea in 0.1 M KOH [22]. An important factor of a supported LDH electrode material is the adhesion of the LDH to the support. If not given by the materials used, binders must be added that are often non-conductive and can increase the electron transfer resistance. The NCW support serves both as an electrical conductor and as a catalyst-docking platform due to its capability to directly anchor the LDH material, as a result of the presence of nitrogen centers. With NCW, there is no need to add an insulating binder. Different molar ratios between Ni and Fe were studied in order to identify the best catalyst. The highest current response was recorded with a Ni to Fe molar ratio of 3:1 due to the most efficient synergistic action of the two metal ions. The sensor fabricated using this Ni/Fe LDH showed a very good stability and reproducibility, and the best sensing performance towards urea (sensitivity of 53 μA mM^−1^ cm^−2^, a linear range from 0.5 to 8 mM, and a limit of detection of 0.114 mM). The sensor exploited Ni^3+^ ions as redox mediators, boosted by the presence of Fe cations, for the electrocatalytic oxidation of urea to N_2_, CO_2_, and H_2_O working at +0.9 V vs. Ag/AgCl (sat. NaCl), and also demonstrated optimal anti-interference properties. Detecting 0.5 mM urea in the presence of 0.1 mM interferents like glucose, UA, and AA, and ionic species, like Na^+^, K^+^, Cl^−^, and F^−^, did not cause a significant increase in the anodic current for urea.

In all the examples described above, it can be noticed that the measurements were performed in alkaline solutions. This is because LDHs generally show a limited stability at lower pH values as the hydroxide ions can be protonated, reducing the material conductivity. Therefore, in order to be able to work at lower pHs, some strategies have been proposed in the literature aiming, for instance, to increase the electrical conductivity of LDHs. As an example, synthesizing hollow structures of Ni/Co LDHs, it was possible to enhance the electron transfer rate of the material, as a result of a very large surface area and the presence of more active sites of Ni and Co ions. In this way, it was possible to operate at moderate pH values, here 7.00, in 0.1 M PBS, for the electrocatalytic oxidation of sumatriptan and naproxen [23].

**Figure 1:**
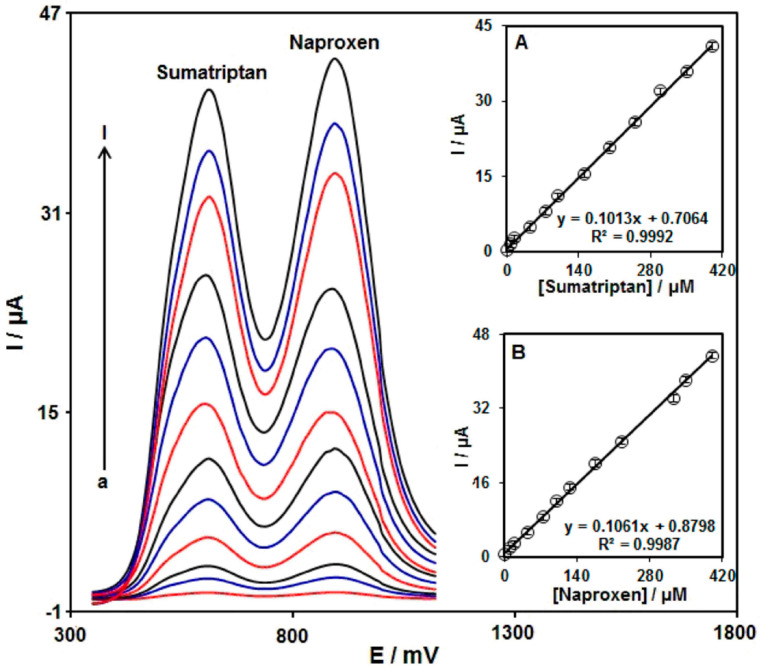
DPVs of Ni/Co LDH/SPE in 0.1 MPBS (pH 7.0) with various concentrations of sumatriptan (a–l: 1.0, 7.5, 15.0, 45.0, 75.0, 100.0, 150.0, 200.0, 250.0, 300.0, 350.0, and 400.0 µM) and naproxen (a–l: 1.0, 10.0, 20.0, 45.0, 75.0, 100.0, 125.0, 175.0, 225.0, 325.0, 350.0, and 400.0 µM). Insets: (A) the plot of peak current versus sumatriptan concentration, and (B) the plot of peak current versus naproxen concentration. Reproduced from ref. [23] (open access).

In order to demonstrate the use of disposable sensors, the Ni/Co LDH was used to modify commercial screen-printed electrodes (SPEs) which were employed to determine sumatriptan, in the presence of naproxen, in pharmaceutical and biological samples. Indeed, satisfactory recoveries were obtained. DPV was used as an analytical technique, which allowed us to record well-separated peaks between the two analytes, as can be seen in Figure 1. The proposed sensor showed a very good selectivity testing 500-fold concentrations of inorganic ions, 300-fold concentrations of fructose, glucose, and lactose, 100-fold concentrations of histidine, phenyl alanine, methionine, and cysteine, and 20-fold concentrations of levodopa and UA, without evidencing any interference in the determination of sumatriptan and naproxen.

### 2.2. Composites Based on LDHs and Carbon Nanomaterials

The electrical conductivity of LDHs can be increased by means of carbon nanomaterials which can be intercalated inside the layered structure. Shangguan et al. chemically synthesized a Co/Al LDH as such, and with reduced graphene oxide (rGO), Co/Al-LDH/rGO, and then the latter material was submitted to an oxidation treatment to obtain Co/Al-OOH/rGO nanosheets. The three LDHs were employed to modify glassy carbon electrodes (GCEs) which were applied successfully for the electrochemical detection of epinephrine (EP) and acetaminophen (AC) in pharmaceutical samples and biological fluids [24]. The measurements were carried out in phosphate buffer (PBS) at pH 7.00 by using DPV, and the Co/Al-OOH/rGO/GCE was found to be the sensor more suitable for the determination of EP and AC, since it displayed a better electrocatalytic activity for the oxidation of the two analytes due to the ability to carry out a faster electron transfer. The sensitivities and detection limits for the determination of EP and AC were 12.2 μA μM^−1^ cm^−2^, 0.023 μM L^−1^, and 4.87 μA μM^−1^ cm^−2^, 0.058 μM L^−1^, and the sensor responded linearly in the concentration range from 0.1 to 25 μM and 0.1–30 μM, respectively. In addition, the sensor selectivity proved excellent since 3 μM solutions of UA and AA, glucose, caffeine, naproxen, alanine, phenylalanine, methionine, and glycine, as well as 300-fold more Na^+^, K^+^, NH_4_^+^, Ca^2+^, Mg^2+^, ClO_4_^−^, SCN^−^, and NO_3_^−^, did not interfere in the EP and AC determination.

Another work describing the cyclic voltammetric (CV) determination of monosodium glutamate (MSG) in potassium nitrate [10] employed a carbon SPE modified with either a Ni_3_Al-CO_3_ LDH film, synthesized by pulse laser deposition, or Ni/Al LDH and Ni/Al LDH + graphene composite films. By combining LDH and G, a composite was obtained where the degree of restacking of graphene flakes was reduced, thus facilitating the charge transfer during the electrode process. The sensitivity performances of the modified electrodes were significantly higher than those of the bare SPEs. The best results were recorded for the graphene composite film, namely, a value of 8.6 ± 0.6 μA cm^−2^ μM^−1^, and, therefore, this composite was chosen as the optimal sensor for MSG.

Exploiting a GCE modified with multiwalled carbon nanotubes (MWCNTs) and a hybrid Ni/Al LDH/GO, a sensor was fabricated for guanine (GUA) and adenine (AD) determination, which was applied to the measurement of the two purine bases in thermally denatured DNA by using linear sweep voltammetry (LSV) at neutral pH. The oxidation peak currents of the analytes were remarkably higher than the ones observed using the bare GCE, and an anticipation of the peak potentials occurred, thus confirming the excellent electrocatalytic performance of the hybrid electrode modifier, due to the synergistic effects of the two carbon nanomaterials [25]

Actually, an exfoliated GO suspension was preliminarily reduced before adding Ni and Al nitrates as LDH precursors into the basic solution, in order to obtain a more conductive rGO. Thereafter, the suspension was submitted to an ultrasonic treatment for 2 h under nitrogen. The TEM and SEM characterizations showed that most of the nanostructured LDH/GO hybrid material was adjacent to the surface of MWCNTs, thus confirming a strong affinity between the two matrices. Furthermore, a selectivity study of the developed sensor was carried out considering a large number of both inorganic and organic compounds. The results confirmed that none of the species interfered significantly in the GUA and AD determinations.

### 2.3. Composites Based on LDHs and Metal Nanoparticles

**Figure 2:**
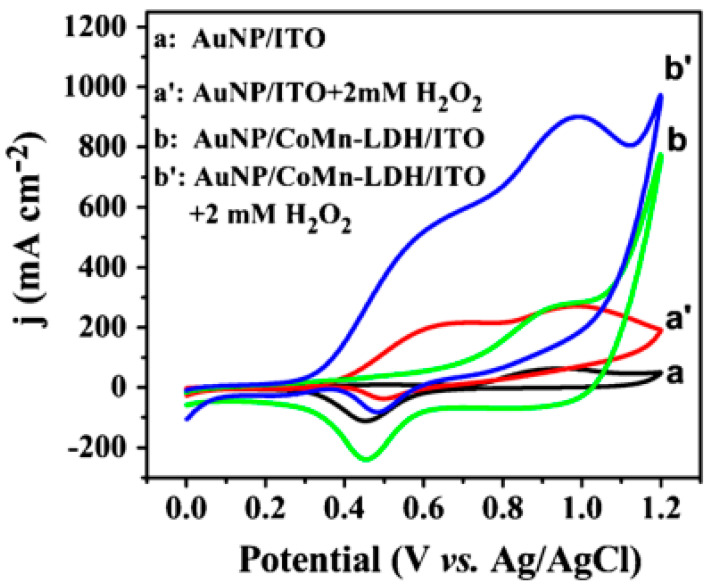
CV scans of different electrodes in 0.1 M phosphate-buffered solution (pH 7.0) in the absence (a, b) and presence (a′, b′) of 2.0 mM H_2_O_2_. Scan rate: 0.1 V∙s^−1^. Reproduced with permission from ref. [27].

Another approach to work with LDHs at lower pHs is based on the enhancement of the LDH electrical conductivity induced by the presence of metal nanoparticles (NPs) instead of using carbonaceous nanomaterials. Some electrochemical sensors based on LDHs coated with noble metals NPs (such as Au, Pt, and Ag) demonstrated excellent electrocatalytic performances [26]. For instance, an electrochemical sensor for H_2_O_2_ was constructed by electrodepositing Au NPs with a typical mean size of 12 nm on an indium tin oxide (ITO) electrode modified with a Co- and Mn-based LDH [27]. In such a case, the catalytic current of the modified electrode was much higher than the one recorded at the Au NP/ITO electrode, and the onset oxidation potential was less anodic. This result supported the idea that the Au NPs, that were deposited on the LDH support, displayed better electrocatalytic performances toward hydrogen peroxide than those that were directly deposited on the ITO electrode (see Figure 2 which refers to CV curves recorded in 0.1 M phosphate buffer (pH = 7.0) in the absence (a, b) and presence (a′, b′) of 2.0 mM H_2_O_2_). Furthermore, also, the electrode modified only with the Co/Mn LDH was proven to exhibit electrocatalytic activity due to the reaction between H_2_O_2_ and the redox-active metals in the brucitic layers, even if much lower than that of the Au NP-modified electrodes. It was previously demonstrated that the integration of mixed-valence transition metals like Co and Mn in the LDH layers can originate a well-organized electronic framework, leading to an increased conductivity [28]. In addition, the presence of manganese ions plays a key role both in anchoring the Au NPs and avoiding their aggregation, and the hydroxyl groups present on the surface of the LDH are beneficial for the interaction with the Au NPs; all these phenomena contribute to enhance the stability and the catalytic efficiency of the composite. At the best working voltage of +0.55 V (vs. Ag/AgCl/sat. KCl electrode), the sensor displayed a wide linear range (0.1 μM to 1.27 mM), a low detection limit (0.06 μM), and a high sensitivity (125.0 μA∙mM^−1^ cm^−2^). These analytical features are superior to those of most previously reported Au NP-based composite modified electrodes, and confirm that Co/Mn LDHs can be considered as optimal supports for the deposition of Au NPs to produce modified electrodes for the development of electrochemical sensors. The sensor was employed to determine H_2_O_2_ levels in human serum samples with the standard addition method with excellent recoveries [27].

Among the metal nanoparticles, nanoscale zero-valent iron (nZVI) NPs are promising for electroanalytical applications as they possess characteristics such as magnetism, electrical conductivity, high surface activity, and optimal electrocatalytic activity. Despite these properties, their effective application is scarce, especially due to their tendency to agglomerate/oxidize, just after the synthesis performed by chemical reduction of Fe^3+^ ion with sodium borohydride, which worsens their performance. The problem can be overcome by using LDHs as support material during the synthesis, due to their excellent exchange and adsorption properties. To this aim, a Ni/Al LDH was chosen and the nanocomposite was employed to modify a boron-doped diamond (BDD) surface to develop a sensor for the determination of the chlorpromazine antipsychotic drug in pharmaceutical formulations and human serum samples by square wave voltammetry (SWV) [29]. The specific surface area of the modified electrode was noticeably increased due to the presence of the nanocomposite, which confirmed that the mesoporous structure of LDH was helpful in limiting the restacking of the nZVI NPs. The modified electrode exhibited excellent electrocatalytic activity for the oxidation of chlorpromazine compared with the bare BDD electrode, working in PBS solution (pH 7.4). The linearity was verified from 0.1 to 8.0 μM with a low detection limit of 0.005 μM. The selectivity studies demonstrated that the sensor displayed no interference from most common compounds, which can be present in real samples, like Ca^2+^, Mg^2+^, Na^+^, K^+^, glucose, lactose, and sucrose, since the oxidation current of chlorpromazine was substantially unvaried.

As far as metal NPs are concerned, it is well-known that their catalytic properties are strongly influenced by their morphology [30]. Dendritic materials are ideal as electrode modifiers due to their high surface area and open porous structure. Among the various synthesis approaches, electrodeposition is a simple and versatile technique for the synthesis of dendrites, which also assures the production of pure films that adhere well to the electrode surface [31].

**Figure 3:**
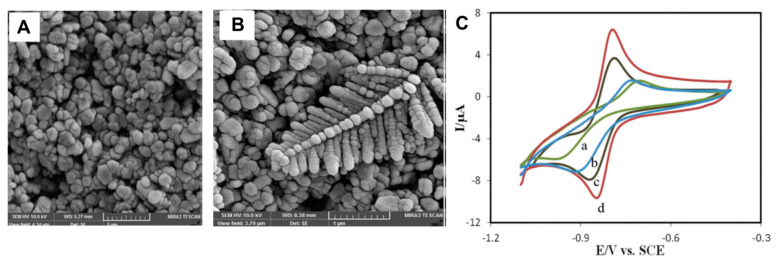
FESEM images at high magnifications of (**A**) LDH/GCE and (**B**) AgND/LDH/GCE. (**C**) CVs of 0.5 mmol L^−1^ PZA at bare GCE (a), LDH/GCE (b), AgND/GCE (c), and AgND/LDH/GCE (d). Supporting electrolyte: 0.1 M PBS (pH 7.0). Reproduced with permission from ref. [5].

Recently, an electrochemical sensor for pyrazinamide (PZA), which is the most active among the four drugs generally employed for the treatment of tuberculosis, has been reported which exploits the electrocatalytic properties of Ag nanodendrites (AgNDs) electrodeposited on a Zn/Al-LDH-modified electrode (Figure 3A,B) [5]. Under the best experimental conditions, the current response recorded by DPV increased linearly with PZA concentrations in a wide range from 9.0 × 10^−7^ to 5.2 × 10^−4^ mol L^−1^, and the detection limit was 7.2 × 10^−7^ mol L^−1^ (S/N = 3). Moreover, the sensor displayed good stability and anti-interference properties. The electrochemical behavior of PZA in PBS (pH 7.0) was investigated by CV over the potential range from −1.1 to −0.4 V (vs SCE) at the bare GCE (curve a), LDH/GCE (curve b), AgND/GCE (curve c), and AgND/LDH/GCE (curve d) (See Figure 3C). At the bare GCE, PZA displayed a quasi-reversible electron process which improved as to both the peak currents and electrochemical reversibility at the LDH/GCE and AgND/GCE. Moreover, at the AgND/LDH/GCE, the two features furtherly increased, thus confirming the noticeable catalytic ability of the modified electrode toward the PZA redox reaction.

This result was ascribable to the synergistic effects of the LDH and Ag nanodendrite composite which were related to the higher surface-area-to-volume ratio, as demonstrated by performing double-layer capacitance measurements of the real surface area. The sensor was applied for the determination of PZA in commercial pharmaceutical tablets, and in spiked human serum and urine samples, and satisfactory results were obtained in all three cases.

### 2.4. Supports for LDHs

**Figure 4:**
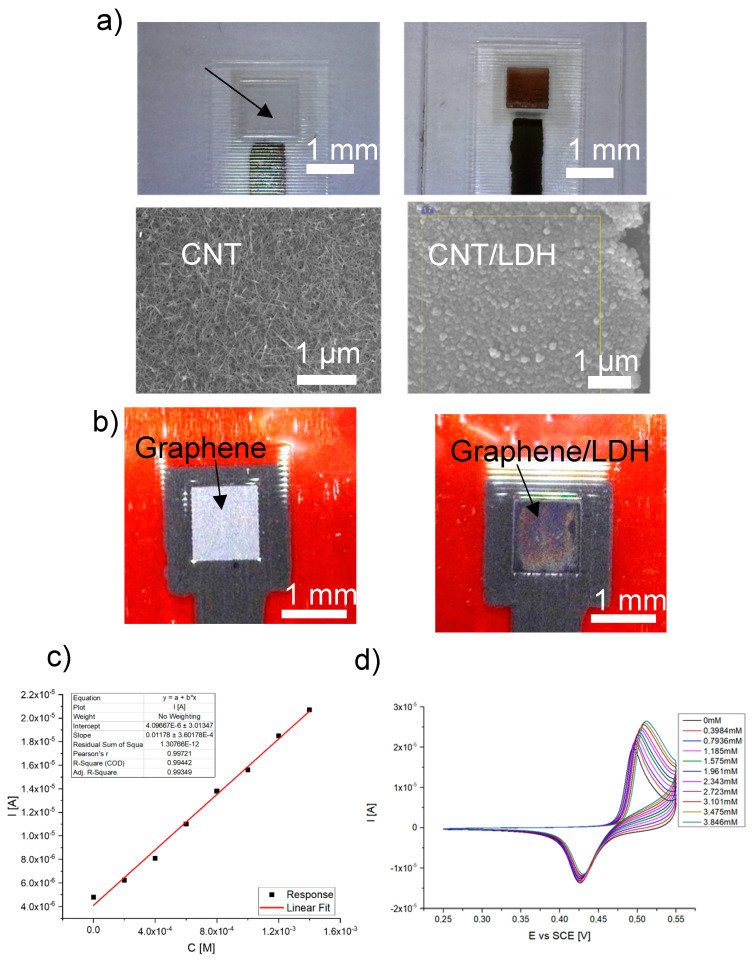
Inkjet-printed carbonaceous electrodes (CNT (**a**) and graphene (**b**)) before and after a potentiodynamic deposition of Ni/Al LDH. (**c**) Calibration graph for methanol in 0.1 M KOH using a Ni/Al LDH/inkjet-printed CNT electrode. (**d**) CVs for the determination of ethanol in 0.1 M KOH using a Ni/Al LDH/inkjet-printed graphene electrode. Unpublished results for discussion.

As demonstrated so far, not only is the selection of the cations of LDHs as the electrode modifier important, but often, also, the LDHs support, generally with the aim to increase the electrical conductivity of the LDH layers. Newer developments focus on the microfabrication of miniaturized low-cost electrochemical sensors entirely based on flexible materials. In the following paragraph, we would like to give an idea about the advantages and disadvantages in printing LDH-based electrochemical sensors according to our experiences. For decades, screen-printing (examples have been included above) has been an established technique to print cheap pastes for sensor production. The geometries of the electrodes are defined by specifically prepared masks containing screens through which the paste is pressed onto a substrate. Nowadays, maskless material deposition techniques are coming more and more into the focus of research, such as inkjet printing [32]. Inkjet printing is based on the ejection of picoliter droplets on demand by creating a pressure pulse inside the nozzles of the printheads, using, for instance, piezoelectric actuation. Inkjet printing is considered as a digital printing technique since computer-generated patterns can be printed without screens, allowing the rapid iterative development of sensor printing. One key advantage of inkjet printing is that the amount of material printed is highly controlled as the mass/area. However, a stable printing process requires us to use inks that fulfill certain rheological parameters, such as surface tension (~30 mN/m) and viscosity (2–10 mPa s). Moreover, the particles in the ink shall be of a lower nanometric dimension and neither agglomerate nor aggregate to avoid irreversible nozzle clogging. The latter is an often underrated issue when research groups plan to print their own nanoparticles. Formulating single-material inks is already challenging by itself, in particular, as carbon nanomaterials like graphene and carbon nanotubes tend to stack easily to each other. Printing, for instance, LDH–carbon composites becomes even more complicated as the mixed-ink components increase the complexity of the fluid. One approach could be, first, to print thin layers of carbon nanotubes or graphene on flexible plastic supports and, then, to deposit a thin layer of LDHs on top. Figure 4a,b show two examples where bare CNTs and graphene electrodes were covered with Ni/Al LDHs by using a potentiodynamic deposition. Electrochemical characterizations demonstrate the stability of the LDH layer. However, it has been noticed that the electrochemical deposition of LDHs seems to occur also inside the carbon layer. In this way, the CNTs and graphene sheets might detach, causing an increased tube-to-tube or sheet-to-sheet resistance. Nevertheless, the sensors can be used for the quantitative determination of methanol or ethanol in alkaline solutions (Figure 4c,d). In an alternative approach, the LDH layer could also be inkjet-printed, making an ink from pre-synthesized Ni/Al LDHs [33]. Therefore, a fully inkjet-printed sensor could be the solution.

## 3. LDHs Not Containing Redox-Active Metal Centers

### 3.1. LDHs Loaded with Redox-Active Molecules

**Figure 5:**
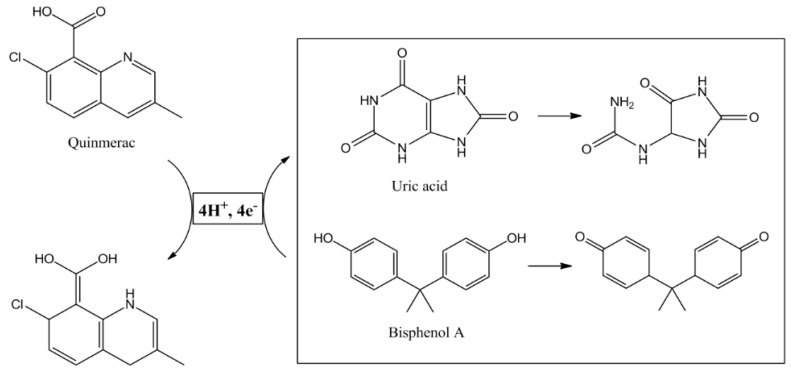
Proposed reaction mechanism of UA and BPA at the surface of modified MWCNT paste electrode. Reproduced with permission from ref. [34].

When the LDHs’ brucitic layers do not contain electroactive cations, they can be used as electrode modifiers for the following reasons: their excellent anion exchange properties, tunability of the interlayer space which can accommodate various guest molecules acting as receptors or aiming to increase the selectivity of a sensor, and great adsorption capability related to their large surface area, which leads to an increased sensitivity for the determination of an analyte. If a guest molecule of the initially redox-center-less LDHs is a redox-active compound, the resulting LDHs behave in a similar way as the ones with initially present redox-active metal centers as discussed vide supra, since it can act as a redox mediator for the electrochemical determination of the analyte of interest. For instance, Nurashikin et al. reported the simultaneous determination of UA and bisphenol A (BPA) by means of a multiwalled carbon nanotube paste electrode (MWCNTPE) which was modified with a Zn/Al LDH intercalated with the quinmerac (7-chloro-3-methyl-8-quinolinecarboxylic acid) herbicide (Zn/Al-LDH-QM) [34]. The LDH was chemically synthesized at a slightly alkaline pH in the presence of the nitrate salts of Al and Zn and the herbicide. The dried precipitate was finely ground, added with different percentages of MWCNTs and some drops of paraffin oil in order to obtain the paste. The Zn/Al-LDH-QM/MWCNTPE displayed a good conductivity with a high electron transfer rate, as demonstrated by EIS and CV investigations, and the key role of Zn/Al-LDH-QM as a redox mediator for the electrocatalytic oxidation of the two analytes was confirmed. The reaction mechanism mediated by quinmerac is shown in Figure 5.

UA and BPA were determined by SWV optimizing several parameters like those relevant to the analytical technique (the best ones were as follows: frequency 170 Hz, step size 4 mV, and pulse size 50 mV), the percentages of MWCNTs and the modifier (the optimal one resulting in 95:5), and solution pH (from 5.4 to 7.4; the chosen value being 6.0). The sensor possessed a wide linear range of UA and BPA concentration (up to 100.0 μM) with a detection limit of 0.065 μM and 0.049 μM, respectively, and was applied to the analysis of real urine and water samples. Furthermore, the modified electrode showed a very good selectivity since there was no interference from ascorbic and glutamic acids, glucose, sucrose, sodium salicylate, phthalate, acetaminophen, captopril, dopamine, and hydroquinone.

In another paper, a voltammetric sensor with a dual response for Fe(III) and dopamine was proposed using a flexible ITO/PET support which was first coated with a GO layer by electrophoretic deposition (thickness of several nanometers) in order to increase the electrical conductivity, and then with Mg/Al LDH nanoplatelets, alternated with an electroactive naphthalimide dye, which was assembled by layer-by-layer (LbL) deposition [35]. The dye, N-ferrocenyl-1,8-naphthalimide, (Fc-NAPH), adsorbed on the electrode surface, emits fluorescence when the electrochemical reaction to (FC-NAPH)^+^ occurs. The proposed electrode could be exploited to detect electroactive analytes as a result of the presence of the ferrocene moiety, which contains a Fe^3+^/Fe^2+^ redox center. The electrode was characterized by CV in neutral PBS, and the anodic and cathodic peaks related to the redox process involving ferrocene were localized at −0.07 V and −0.315 V vs. Ag/AgCl, respectively. If Fe^3+^ is present in solution, being an oxidizing ion, it can oxidize the Fc to Fc^+^ with a consequent increase in the fluorescence emission. At the same time, the (Fc-naphthalimide)^+^ concentration is also increased, and a higher cathodic current than the anodic one is recorded performing CV. The cathodic peak current showed a linear dependence with the increase in ferric ion concentration in the range of 0.5 × 10^−6^–8 × 10^−5^ M, with a detection limit equal to 0.03 µM.

The dual electrode was also used to determine very low concentrations of dopamine (DA) due to the capability of Fe^3+^ to form a strong chelate with DA; in such a way, the fluorescence emission decreased in the presence of DA molecules and the oxidation peak of Fc in the dye increased. Both electrochemical and optical signals could be used to detect DA with a high sensitivity; in the CV-based method, a detection limit of 0.06 nM and a linear range from 1.0 × 10^−10^ to 1.5 × 10^−8^ M were obtained. The results of the study demonstrated that the (NALD-5)-modified electrode could be very efficient in detecting nanomolar DA levels in real pharmaceutical samples. Furthermore, the selectivity yielded a very good result since DA could be successfully determined also in the presence of the most commonly encountered interferents when biological samples are analyzed, i.e., UA and AA. Finally, the molecular design of the sensor, which was based on several nanometric layers of a conductive network coating the ITO/PET support, allowed for a rapid direct electron transfer which is essential for a speedy sensing assay, as required for the in vivo monitoring of neurotransmitters.

The LDHs’ high specific surface area can serve for the immobilization of some molecules with a negative charge and electroactivity; if these molecules are capable of acting as mediators for the electrocatalytic reduction of the analytes, the modified electrode can also be employed for the determination of reducible compounds.

To this aim, nickel hexacyanoferrate Ni_2_[Fe(CN)_6_], shortly NiHCF, has been widely used for analytical applications as a result of the presence of the Fe(III)/Fe(II) couple, which is easily involved in a reversible redox reaction [36]. A sensitive and selective device has been developed by electrodepositing NiHCF on a gold support coated with a Ni/Al LDH for the determination of doxorubicin (DOX) at neutral pHs in human blood serum samples. The measurement was performed by DPV under a nitrogen atmosphere (potential range from −0.5 V to −0.8 V, vs. Ag/AgCl/saturated KCl), and the response was linear with the DOX concentration in the range 1.0 × 10^−8^–6.2 × 10^−6^ M, with a limit of detection of 1.9 × 10^−9^ M, and an excellent sensitivity of 14.71 A mol L^−1^ cm^−2^. This result was ascribable to the large increase in the electrochemically active surface area (ECSA) due to the presence of NiHCF. It was calculated by CV and had a result of 0.5833 cm^2^, i.e., much higher than the values displayed by the bare Au (0.0246 cm^2^) and the Ni/Al–LDH/Au electrode (0.1509 cm^2^). The fabricated sensor also displayed good reproducibility, anti-interference, and long-term stability properties [14].

### 3.2. LDHs Loaded with Anionic Chelating Agents

By substituting the LDHs’ interlayer anion with a chelating agent, the detectability of metal ions can be accomplished. Mercury is well-known to be highly toxic, even at low levels, because of its reactivity, volatility, and solubility in water and living tissues.

MWCNTs modified with a Zn/Al LDH intercalated with 3(4-hydroxyphenyl)propionate (Zn/Al-LDH-HPP) were employed to prepare a carbon paste electrode for Hg^2+^ determination by CV. The responses of 2.0 × 10^−4^ M Hg(II) in KCl (pH 5.0, acetate buffer) showed that the electrode process involving the Hg(II)/Hg redox couple only occurred if the CNTs were modified with the LDH, thus supporting the formation of a complex between Hg ions and 3(4-hydroxyphenyl)propionate. The anodic peak was centered at 93.9 mV (vs saturated Ag/AgCl) whereas the cathodic peak was centered at −37.9 mV [37]. Under the best experimental conditions, the sensor gave a linear response within the concentration ranges of 1.0 × 10^−9^–1.0 × 10^−7^ M and 1.0 × 10^−7^–1.0 × 10^−3^ M, and the limit of detection for Hg(II) was 5.0 × 10^−10^ M. The selectivity was tested against the most common metal ions at a 25-fold concentration, finding out that none interfered on the determination of Hg(II) with the exception of Zn(II). The proposed carbon paste electrode was successfully applied to determine Hg^2+^ in real samples such as fish and shellfish.

Another electrochemical sensor based on the thioglycolic-acid-intercalated Mg/Al LDH (Mg-Al-TGA LDH)-modified electrode was reported for the determination of trace mercury levels using square wave anodic stripping voltammetry (SWASV). Moreover, in this case, the chelating properties of the anion inserted in the interlayer gallery by an exchange reaction were responsible of the electrochemical response towards Hg^2+^ [38]. Several variables affecting the stripping peak current were investigated in order to optimize the current value. Under the best conditions, the sensor displayed an excellent anti-interference ability, a wide linearity range (2.0–800 nM) with a detection limit of 0.8 nM (S/N = 3), which is below the values given by the World Health Organization for the drinking water. It was applied to the determination of Hg(II) in water samples, obtaining satisfactory recoveries.

A recent paper describes the employment of a GCE modified with EDTA-Mg/Al LDH for the sensitive detection of Fe(II) by DPV over an applied potential range from −0.2 to 0.2 V, vs. Ag/AgCl/sat. KCl [39]. The determination of a very low level of ferrous ions is crucial since the iron unbound forms are dangerous for vital organs like the heart, kidney, and liver [40]. EDTA was intercalated into the LDH galleries by an exchange reaction substituting the original chloride ion. Again, the EDTA ability to chelate Fe^2+^ was responsible of the analytical signal which was related to the oxidation of Fe^2+^ to Fe^3+^, carried out in KCl solution (pH 7.0). The developed electrode demonstrated an excellent selectivity for Fe^2+^ determination, since various metal ions, commonly encountered within the human body, including Zn^2+^, Cu^2+^, Ni^2+^, Co^2+^, Mn^2+^, Mg^2+^, K^+^, Na^+^, and Cu^2+^, did not alter appreciably the current recorded in the presence of only Fe^2+^. Moreover, the EDTA-Mg/Al LDH GCE exhibited a fast response time (~45 s), a wide concentration linearity range (0.1–102.1 μM), and a low detection limit of 50 nM. The practical applicability of the developed modified electrode was tested on an artificial blood serum sample [34].

Taking advantage of the easy exchangeability of the interlayer anion, the hydrophilicity of LDHs can be modified in order to enhance the adsorption affinity toward nonionic organic compounds, which is generally poor in water. Inserting anionic surfactants, such as aliphatic carboxylates, organic sulfates, and organic sulfonates, among the layers of the LDHs, it is possible to synthesize organophilic LDHs (org-LDHs). GCEs were coated with a Mg/Al LDH intercalated with dodecyl sulfate (SDS) to favor the accessibility of paracetamol, and, later, with Au NPs to electrocatalyze the oxidation of the analyte [41]. A DPV analytical method was developed for the simultaneous determination of paracetamol, 4-aminophenol, which is its metabolite, and dopamine, at pH 7.0 using the modified GCE.

It exhibited excellent redox activity, with an increased oxidation current and a minor overpotential with respect to those recorded at the bare GCE, the Au NP-modified GCE, and the SDS-LDH-modified GCE. The fabrication procedure was very simple, rapid, and cheap. The sensor possessed good stability and anti-interference performance, since only UA displayed a significant interference. The response was linear for paracetamol concentrations ranging from 0.5 to 400 μM, with a detection limit of 0.13 μM (S/N = 3). The modified electrode was practically applied to the determination of paracetamol and dopamine, and of paracetamol and 4-aminophenol, respectively, in pharmaceutical tablets and in spiked human serum samples.

### 3.3. LDHs Acting as Analyte Preconcentrators

**Figure 6:**
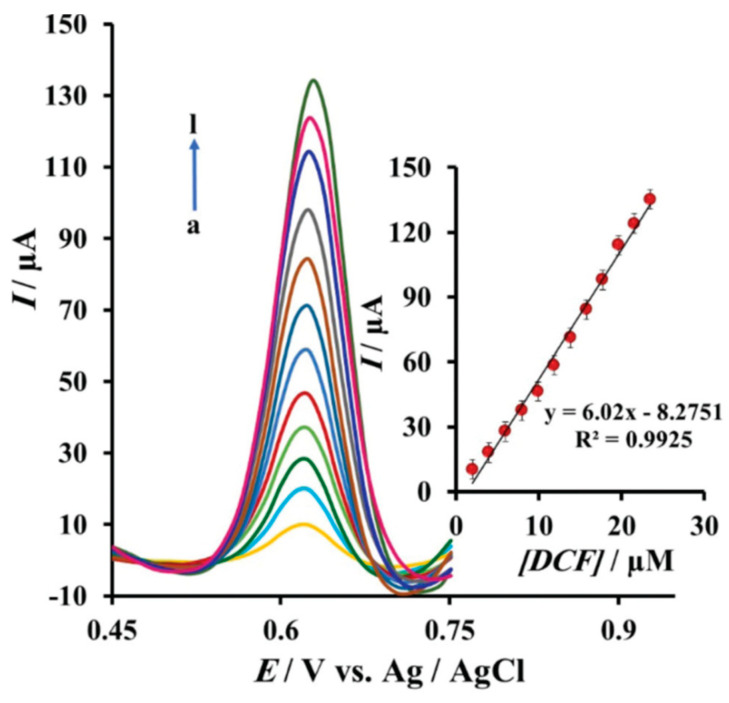
DPV responses of the Cu/Al LDH/CFYM/GPGE in PBS (pH 7.0) solution at a scan rate of 10 mV s^−1^ with different concentrations of DCF from (a) to (l) are 1.99, 3.98, 5.96, 7.94, 9.9, 11.86, 13.81, 15.74, 17.68, 19.61, 21.53, and 23.44 mM. The inset shows the relationship of current responses to DCF concentration. Reproduced with permission from ref. [43].

Based on the LDHs’ structure with a permanent positive charge in the brucitic layers, these clays possess a high anion exchange capacity which makes them suitable as substitutes of the most common anion exchangers both for the sensing and removing of environmental pollutants [42]. Recently, a sensitive and simple determination of diclofenac sodium salt (DCF) was proposed by exploiting this feature. The authors developed pencil graphite electrodes which were graphenized by an oxidation treatment at 2 V vs. Ag/AgCl, in sulfuric acid, in order to form GO nanoparticles on their surface (GPGE) [43]. Later, they were modified with a mixture of a Cu/Al LDH and a chicken feet yellow membrane (CFYM) at an optimized weight ratio. CFYM is made up of a biopolymer containing carbohydrates and amino acids, like glycine and arginine, which was demonstrated to be an excellent adsorbent for preconcentrating organic pollutants [44]. The Cu/Al LDH/CFYM/GPGE-modified electrode showed a better electrochemical response toward DCF oxidation than the unmodified GPGE both using cyclic voltammetry and differential pulse voltammetry, working in PBS, pH 7.0. These results could be explained by the ECSA values determined by CV, which were 0.068 cm^2^ for the unmodified PGE, 0.174 cm^2^ for GPGE, and 0.484 cm^2^ for the Cu/Al LDH/CFYM/GPGE. Furthermore, the nanocomposite made of LDH and CFYM possesses a great ability to preconcentrate DCF and this property has to be taken into account to explain the high sensitivity of the sensor. Figure 6 shows the DPV curves recorded in the presence of increasing DCF concentrations together with the relevant calibration line in the range of 2–23.5 mM. The LOD and LOQ values were calculated as 1.9 µM and 6.4 µM, respectively. Moreover, the anti-interference ability of the sensor was checked by analyzing the following compounds, in the presence of 10 µM DCF: ascorbic acid, l-cysteine, citric and oxalic acids, glucose, sucrose, lactose, fructose, urea, K^+^, Na^+^, and Cl^-^ [43].

The applicability of the nanocomposite-modified GPGE was demonstrated, determining DCF in pharmaceuticals and biological real samples by DPV with satisfactory recoveries.

Based on Zhao’s observations [45] that GC electrodes modified with Ag NPs and proline displayed a lower negative potential and higher current densities towards the reduction of H_2_O_2_, a voltammetric sensor exploiting a Zn/Al-LDH-modified GC electrode was developed for the determination of hydrogen peroxide in healthy and diabetic human urine [46]. The logic behind its operation is the catalytic activity exerted by LDH for the decomposition of H_2_O_2_ to oxygen and water, and then the measurement of the reduction current of the produced oxygen at the GC-LDH electrode, biased at −0.58 V vs. Ag/AgCl/Cl^−^. The best performance of the sensor was obtained when proline was present in PBS at pH = 7.0. At this pH, which is higher than the isoelectric point of proline (6.3), the amino acid is largely present in the anionic form, and, therefore, can be easily preconcentrated inside the LDH interlayers, thus favoring the catalytic activity of the LDH towards H_2_O_2_. Furthermore, the sensor selectivity was evaluated towards all the most common antioxidant substances found in samples of urine, highlighting no interference effect, whereas only very strong oxidizing species, rarely present in real biological samples, could affect the sensor response.

## 4. Conclusions and Future Perspectives

Layered double hydroxides are promising materials in the field of electroanalysis due to the high surface area, low cost, low toxicity, ion-exchange capability, and easy preparation, as demonstrated by their use in many relevant electroanalytical applications as demonstrated herein. In addition, these materials can possess specific features by tuning their chemical composition or morphology. For instance, the presence of a cation that can be involved in a reversible redox process within an applied potential range is an essential requirement when the LDHs must act as a redox mediator for the electrocatalytic determination of an analyte. The oxidation of the metal centers occurs more effectively in basic solutions. In fact, the OH^−^ concentration has a strong influence on the overall conductivity of LDHs, which increases as the pH is increased, whereas, for low OH^−^ concentrations, the electrochemical processes are inhibited.

The relatively low electrical conductivity could limit the use of LDHs as electrode modifiers for the development of voltammetric sensors, but the drawback can be easily overcome by producing composites with metal NPs or carbon-based nanomaterials, like G or CNTs, which allow for a better electrocatalytic effect, selectivity, and sensitivity. Furthermore, producing these composites is helpful in increasing LDHs’ stability which can be employed also at neutral pHs. In addition, the role of the intercalated anions is essential since they modulate the interlayers spaces so as to accommodate different guest molecules which can serve as receptors or redox mediators. In the case where they display chelating properties, the determination of cations becomes possible.

Regarding the approach of modifying supports with LDHs by coating techniques, the most common one is based on drop-casting. It does not guarantee a good control over the film thickness and a good adhesion between the modifier and the electrode. Fortunately, in the case of LDHs, the modification of any conductive surface can be performed by electrosynthesis, which occurs in a few minutes and allows us to obtain a well-adhered film. In addition, the possibility of easily expanding the interlayer region of LDHs in order to intercalate an active component and of delaminating these materials into single positively charged nanosheets, which can be electrostatically self-assembled, offers perspectives for the design and manufacturing of new electrode surfaces exploiting multifunctional LDH films.

In order to extend the applicability of electrochemical sensors, the electrode material and the kind of sensing device play a key role. During the recent years, most of the usual electrodes based on noble metals or carbon materials have been progressively substituted with more economical systems displaying good electrical properties, high electrochemical reactivity, good mechanical resistance, and ease of fabrication at an industrial level. An example is given by pencil graphite electrodes which also display a good accessibility to the analyte. As far as the kind of device is concerned, screen-printed electrodes, especially the carbon-based ones, have been considered very suitable for an in situ analysis as a result of their low power requirement, quick response, high sensitivity, and ability to operate at room temperature. Furthermore, they need small volumes of samples, and, consequently, they consume low amounts of reagents. The main drawback of SPEs is related to their fabrication which require several steps, and therefore, they generally display a noticeable variability. The most recent developments in electroanalytical chemistry focus on the microfabrication of miniaturized low-cost electrochemical sensors entirely based on flexible materials. In our opinion, the future in this research field could be focused on maskless material deposition techniques, based on drop-on-demand ink delivery systems, such as inkjet printing. The major issue to face is the stability of inkjet-printable metal nanoparticle inks which can be reached, preventing nanoparticle agglomeration to avoid nozzle clogging, and properly selecting the viscosity, as well as the surface tension, so as to eject stable droplets. Fortunately, nowadays, the transfer of a printing process from a small lab-scale printer to large-scale industrial platforms has become very efficient, so that inkjet printing as mass production technique is foreseen to be applied also at the industrial level even for printing complicated fluids, such as those based on LDH-nanocarbon composites.

## Data Availability

No new data were created or analyzed in this study. Data sharing is not applicable to this article with the exception of the section concerning the inkjet printing technique for which further inquiries can be directed to the corresponding author/s.
